# In Vitro Study on the Adhesive Performance of Some Resin-Based Materials Used to Restore Class II Cavities

**DOI:** 10.3390/ma14154299

**Published:** 2021-07-31

**Authors:** Andreea Ciurea, Ada Gabriela Delean, Andreea Didilescu, Monica Monea, Alexandra Roman, Marius Boariu, Ștefan Ioan Stratul, Camelia-Manuela Mîrza, Iulia Cristina Micu, Petra Șurlin, Andrada Soancă

**Affiliations:** 1 Department of Periodontology, Faculty of Dental Medicine, Iuliu Haţieganu University of Medicine and Pharmacy Cluj-Napoca, 15 Victor Babeş St., 400012 Cluj-Napoca, Romania; andreea_candea@yahoo.com (A.C.); i.cristina.micu@gmail.com (I.C.M.); andrapopovici@gmail.com (A.S.); 2 Department of Odontology and Endodontics, Faculty of Dental Medicine, Iuliu Haţieganu University of Medicine and Pharmacy, 33 Moților St., 400012 Cluj-Napoca, Romania; adadelean@yahoo.com; 3 Department of Embryology, Faculty of Dental Medicine, Carol Davila University of Medicine and Pharmacy, 8 Eroii Sanitari Boulevard, 050474 Bucharest, Romania; andreea.didilescu@umfcd.ro; 4 Department of Odontology and Oral Pathology, George Emil Palade University of Medicine, Pharmacy, Science and Technology, 38 Gheorghe Marinescu St., 540139 Târgu Mureș, Romania; monea_monica@yahoo.com; 5 Department of Restorative Dentistry and Endodontics, Faculty of Dental Medicine, Victor Babeș University of Medicine and Pharmacy, 9 Bd. Revoluției 1989 St., 300070 Timisoara, Romania; boarium@yahoo.com; 6 Department of Periodontology, Faculty of Dental Medicine, Victor Babeș University of Medicine and Pharmacy, 9 Bd. Revoluției 1989 St., 300070 Timisoara, Romania; sbs@online.ro; 7 Department of Pathophysiology, Iuliu Hațieganu University of Medicine and Pharmacy, 2-4 V.Babeș St., 400012 Cluj-Napoca, Romania; camelia.mirza@umfcluj.ro; 8 Department of Periodontology, Faculty of Dental Medicine, University of Medicine and Pharmacy, 2 Petru Rares St., 200349 Craiova, Romania; surlinpetra@gmail.com

**Keywords:** microleakage, composite resin, ormocer, adhesion, tooth

## Abstract

The study aimed to evaluate the adhesive performances of two ormocer materials and two micro-hybrid composites placed to restore class II cavities. We tested the null hypothesis, which considered that the adhesive behaviors of tested materials did not differ. On each extracted tooth, two class II cavities were prepared having an enamel located cervical margin and a cementum located cervical margin, respectively, and were restored using two different restoration techniques. The teeth followed a tooth impregnating protocol and were sectioned and evaluated by optical microscopy to highlight the marginal microleakage around restorations. Cervical and occlusal microleakage as well as microleakage ratios were calculated. The microleakage test showed that all tested materials exhibited some degree of dentinal microleakage both on cervical and occlusal areas irrespective of the restoration technique. Some significant differences were recorded in adhesion performance of the materials. The cervical microleakage ratio was significantly increased for one of the micro-hybrid resin composites in comparison with one of the ormocer materials (*p* = 0.0159). Significantly differences were observed in occlusal microleakage ratios when the two micro-hybrid composites were compared (*p* = 0.047). The results failed to reject the null hypothesis. The present study could not demonstrate the superiority of ormocer-materials relative to conventional composites.

## 1. Introduction

Resin-based composites are the most used materials for direct restorations on both anterior and posterior teeth due to their adequate aesthetics and physico-chemical and biological properties [1]. They are the first restorative treatment choice for both clinicians and patients. As such over 640 million posterior resin composite restorations were placed globally only in 2015 [2]. A meta-analysis on posterior resin composite restorations reported that by 2025, 32 million of these restorations will be replaced due to fracture or repaired due to noticeable wear [3].

The polymerization shrinkage and the consequent stress accumulated at the adhesive interface still represent a major drawback associated with resin composite restorations, especially in posterior regions, due to the uncertainties related to the cervical location of the cavity and technical difficulties [4]. Polymerization stress induces microleakage along the dental–restorative interface, which allows fluid and biofilm penetration and favors decay development, postoperative sensitivity, pulpal pathologies and finally results in failure of the restoration [5]. Posterior resin composites shrink between 2.6% and 7.1% by volume [6]. The extent of shrinkage is highly dependent on the filler load, and the treatment technique of filler particles [7]. Therefore, the properties of direct restorative filling materials require further improvement through compositional modifications in order to fulfil clinical requirements. 

Recent advances in resin-based restorative materials targeted the improvement of their properties by reducing the filler size. More filler particles increase the strength and modulus of elasticity and reduce polymerization shrinkage. Nanocomposites contain sub-micrometer particles (nano fillers), which improve particle distribution, filler loading [8] and their polymerization behavior [7].

Recent bulk fill variants of resin composites were developed in order to simplify the restorative technique and to reduce working time by being placed as increments up de 5 mm [9]. Bulk fill resin composites have some innovative composition adjustments made to reduce shrinkage stress associated with higher curing volume, which rend them predominantly more translucent [10].

Further improvements of organic components of resin-based materials proposed Bis-GMA-alternative monomer compositions. A new generation material named ORMOCER (Organic-Modified Ceramic) was developed by manufacturers. Ormocer materials consist of inorganic–organic co-polymers having less polymerization shrinkage than conventional Bis-GMA-based composites [7] due to the larger three dimensional cross-linked ceramic polysiloxane monomer [11]. The volumetric polymerization shrinkage of ormocer materials was reported to be <2%, thus indicating a better marginal integrity [11]. The first-generation of ormocer materials did not reach the initial expectations as no differences in comparison with conventional composites could be reported [12]. There is some evidence of poorer long-term clinical behavior of restorations carried out with ormocer materials compared to conventional composites [12], but other reports have provided opposite results [10].

The microleakage tests offer useful information regarding the adhesion of restorative materials. Different techniques have been developed in order to evaluate the leakage around the restorations. In vitro tests include the use of dye solutions, radioactive isotopes, air pressure, bacterial activity investigation, scanning electron microscopy or micro-computer tomography investigations [13].

The coloration of the microleakage areas using different dying solutions is the most frequent technique used [13], allowing an easy comparison between studies. The method of dye penetration targets to color the microleakage areas and uses contrasting dying solutions such as 0.2–0.5% basic fuchsin, 50% silver nitrate or 2% methylene blue solutions [13].

The aim of this in vitro study was to evaluate the adhesive performances of two ormocer materials and two micro-hybrid composites placed by two different techniques to restore class II cavities by measuring the cervical and occlusal enamel microleakage after basic fuchsin dye penetration. We tested the null hypothesis, which considered that the adhesive behaviors of restorative materials did not differ.

## 2. Materials and Methods

### 2.1. Ethics and Study Design

The study was done in the Periodontology Department, the Dental Materials Department and the Tissue Engineering Research Centre from Iuliu Haţieganu Medicine and Pharmacy University, Cluj-Napoca. Extracted teeth were obtained after an informed consent from patients according to a protocol approved by the Ethical Board of the Iuliu Haţieganu University (No. 56/3 February 2017). Tooth collections were prepared and preserved in accordance with relevant guidelines and regulations, as detailed elsewhere [14].

Four resin-based materials (two ormocer materials and two micro-hybrid composites) and five groups of experimental teeth were used. On each tooth, two class II cavities were prepared having an enamel located cervical margin and a cementum located cervical margin, respectively. The bulk fill ormocer restored the first group of teeth (group I) with bulk fill technique. After that, each experimental material was used to restore a group of teeth (group II-IV) using the proximal wall technique. The teeth followed a tooth impregnating protocol and were sectioned and evaluated by optical microscopy to highlight the marginal microleakage around restorations. 

### 2.2. Resin Composites and Adhesive Systems

Two ormocer materials, one bulk fill type ormocer (Admira Fusion X-tra^®^ /Voco, Cuxhaven, Germany) (AX) and one conventional ormocer (Admira Fusion^®^ /Voco, Cuxhaven, Germany) (AF), as well as two micro-hybrid composite resins, Essentia^®^ (GC Europe N.V., Leuven, Belgium) (ES) and G-aenial^®^ (GC Europe N.V., Leuven, Belgium) (GE) shade A2, were used to restore class II cavities. The ormocer-based materials were used with the adhesive system Futurabond^®^ M+ (Voco, Cuxhaven, Germany) and the hybrid composites with the G-Bond™ (GC Europe N.V., Leuven, Belgium) (Table 1).

### 2.3. Tooth Restoration and Preparation of Study Groups

Twenty-five third molars free of any lesions were divided in five groups with five teeth per group. Two standard class II cavities were prepared on each tooth, following a former protocol [14] using round diamond-coated burs (Komet 6801.314.018, 5801.314.016, black color coded, Lemgo, Germany), cylindrical-diamond-coated burs (Komet 6837.314.014) mounted on a high speed handpiece with air and water cooling (Figure S1A).

**Figure 1:**
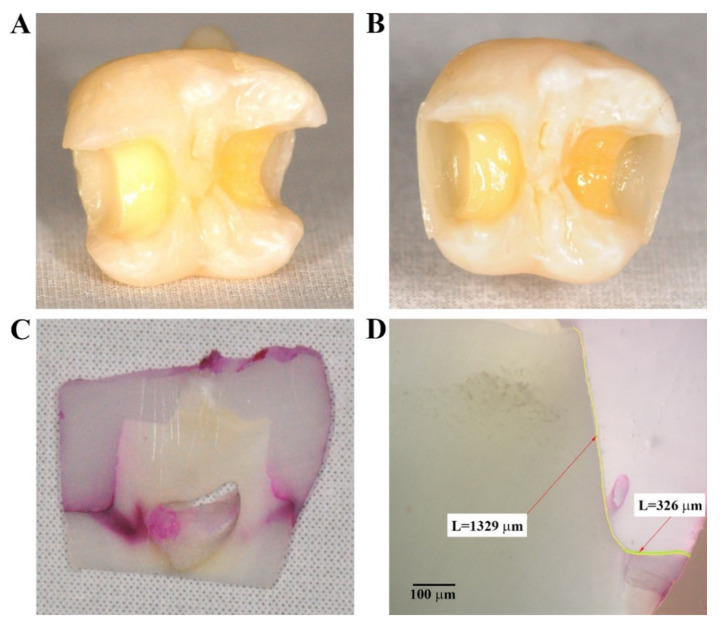
Phases in the preparation of the samples and microleakage evaluation. Class II cavities (**A**); prepared proximal walls (**B**); section containing two AF restorations—dye infiltration in cervical areas (**C**); optical microscopic image of a sample containing a cervical AF restoration—microleakage in dentin (40×) (**D**). Arrow = length (μm).

One proximal cavity of each tooth received a cervical margin located 1 mm coronal to the cement-enamel junction (CEJ) (enamel-located cavity) and the other proximal cavity received a cervical margin located at CEJ (cementum-located cavity) (Figure 1A). The occlusal cavity had a buccal-oral width and a depth of 3 mm, and the proximal cavity had a buccal-oral width of 3 mm, an occluso-cervical dimension about 6 mm, and an mesio-distal depth of the gingival wall of 2 mm. The occlusal enamel margins were not beveled.

The group I—AXB includes teeth restored with AX material using the bulk fill technique. For the other groups, *the proximal wall technique* was used in association with experimental materials and the groups were noted as follows: group II—AF, group II—ES, group III—GE and group V–AX.

The SuperMat™ universal dental matrix tensioning system no. 2181 (Kerr, Bioggio Switzerland) and steel posterior matrices assured a standardised proximal wall reconstruction for both restorative techniques.

The restoration protocol for bulk technique used a first composite layer of 4 mm depth applied on the cavity cervical wall and adapted with a medium size plugger, and then light cured from occlusal direction for 20 s using Demetron A2 light-curing unit (Kerr, Middleton, WI, USA) wavelength 450–470 nm and light intensity of 1000 mW/cm^2^. A second layer of material completed the cavity.

Proximal wall restoration technique is detailed elsewhere [18]. Briefly, a 0.5 mm thin proximal wall was formed and then light-cured, which allowed for the removal of the matrix system (Figure 1B). The remaining cavity was then restored in a centripetal direction.

All the restorations were finished and polished using flame-shaped diamond burs (Komet 8368.314.023, red color coded, Lemgo, Germany) and polishing disks (OptiDisc^®^, Kerr, Bioggio, Switzerland).

### 2.4. Microleakage Test

The protocol is detailed elsewhere [14]. After immersion in 0.5% basic fuchsine solution for 24 h (Figure S1C), the teeth were embedded in acrylic resin (Duracryl^®^ Plus, Spofa Dental, Markova, Czech Republic) and sectioned in the mesiodistal direction using a low-speed diamond saw (Isomet, Buehler Ltd., Lake Bluff, IL, USA) (Figure S1D) resulting in sections 1 mm thick (Figure 1C and Figure S1E). For each tooth, the two central sections (coded sections A and B) were analyzed. For each section, the restoration with the cervical margin located in enamel was coded as A1 or B1, and the restoration with the cervical margin located in cementum was coded with A0 or B0. The microleakage was evaluated with an inverted microscope (Olympus KC301, Olympus America, Inc., Los Angeles, CA, USA) at 40× magnification and was recorded using QuickPhoto Micro 2.2 software (Olympus, Inc, Promicra, Prague, Czech Republic). The extent of microleakage at the tooth-resin interface was quantified using a method previously developed by our team [14,19,20] as variant of a gap measurement approach previously described [21].

A good adhesion was considered the continuous adhesive layer, without dye penetration along the restorative material–tooth interface. The microleakage means the diffusion of the dye along the adhesive interface observed as a pink line on the microscopic images. The microleakage measurements followed the pink penetration lines and were marked by the software in different colors according to their locations. Microleakage measurements (in μm) included the length of dye penetration at cervical level (MLKd marked with a green line), length of dye penetration at occlusal enamel level (MLKe marked with a blue line), and the total length of the tooth–restoration adhesion interface (AIL marked with a yellow line) (Figure 1D). The microleakage proportion was calculated.

### 2.5. Data Analysis

Ten sections per each study group were available for statistical analysis. The Shapiro–Wilk test was used to test the normal distribution, and the variance was tested with the F test. In order to summarize the distribution of quantitative variables, the mean ± sample standard deviation (SD) was used. The comparison of two groups in relation to the quantitative characteristics was performed by Student-t and Mann–Whitney (U) or Wilcoxon tests.

The Pearson correlation coefficient (r) or the Spearman rank correlation coefficient (ρ) were used to detect the correlation between two variables, and the interpretation of the values was done using Colton’s empirical rules.

The significance threshold for the tests used was α = 0.05 (5%).

Statistical data analysis was performed using StatsDirect software (v.2.7.2) (StatsDirect Ltd., Birkenhead, Merseyside, UK). The graphical representation of the results was done with the Excel application (from the Microsoft Office 2010 package).

## 3. Results

After restoring 50 class II cavities on 25 extracted molars, tooth immersion in dye solution and sectioning, the microleakage along the restoration–tooth interface was evaluated on 50 sections each of it containing two restorations. A total of 100 half-sections were examined, and the microleakage ratio at the cervical level as well as at the occlusal level of the restorations were calculated.

### 3.1. Microleakage Measurements

**Figure 2:**
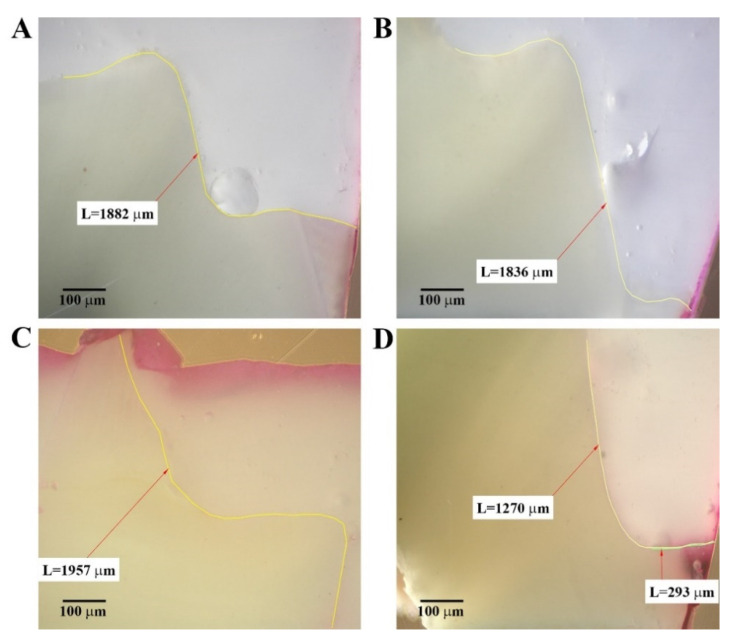
Adhesion of some experimental materials. Good adhesion in cervical area of enamel-located cavities restored with AX by bulk fill technique (group I–AX B) (**A**) and proximal wall technique (group V–AX) (**B**); good occlusal adhesion of an ES restoration (group III—ES) and (**C**); cervical microleakage of an ES restoration in a cementum-located cavity (**D**). Arrow = length (μm).

**Figure 3:**
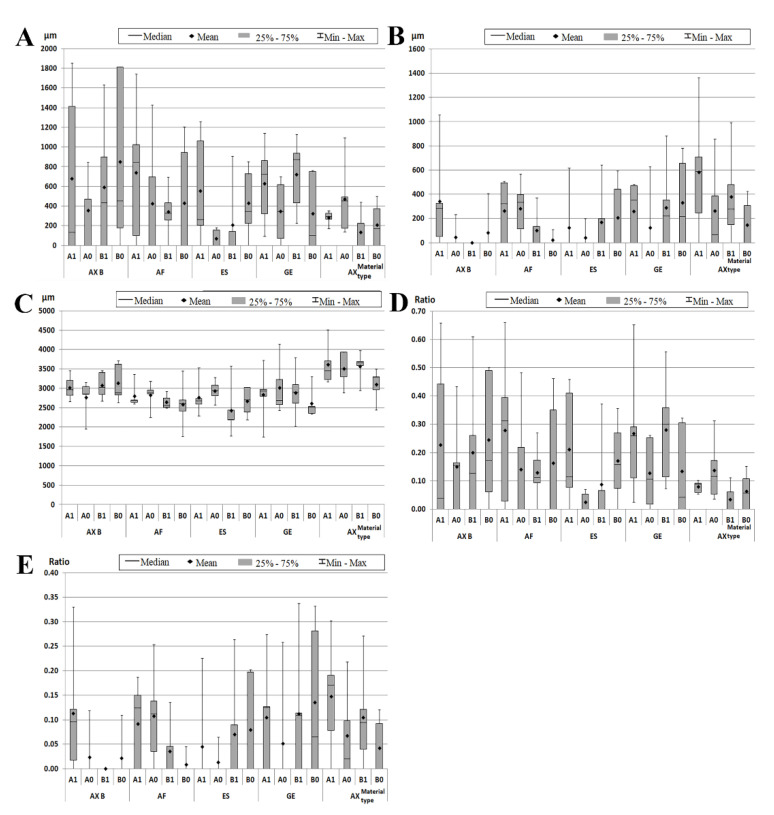
The median and range values of the evaluated parameters in the five groups of restorations, for both types of cavities. Microleakage in cervical area (MLKd) (**A**); microleakage in occlusal area (MLKe) (**B**); total adhesion interface (AIL) (**C**); ratio of cervical microleakage (**D**); and ratio of occlusal microleakage (**E**). Groups I-V restored with AX B = Admira Fusion X-tra applied by bulk fill technique, AF = Admira Fusion, ES= Essentia, GE = G-aenial, AX = Admira Fusion X-tra; A1, B1 = part of the sections containing enamel-located restorations; A0, BO = part of the sections containing cementum-located restorations.

A good adhesive interface was observed for some sections, but most of the restorations presented microleakage both at the cervical and occlusal enamel margins except for some sections that had no leakage (Figure 2). The evaluated parameters associated with the five groups of restored teeth, for both types of restorations (cervical enamel- and cementum-located), are shown in Figure 3, and the significance of the inter- and intra-group comparisons of the parameters are provided in Table S1. No significant differences were observed between sections of different groups in terms of MLKd and MLKe except for a few situations. A significantly increased MLKd was observed for group IV versus group III and for group IV versus group V for B1 sections (*p* = 0.0476 and 0.0317, respectively) and for group V versus group III for A0 sections (*p* = 0.0476). Sections were rather uniform regarding MLKe except for the sections B1 where the values were significantly increased for group V versus group I (*p* = 0.0476) (Table S1).

The highest mean value of MLKd for the enamel-located restorations (sections A1 and B1) was recorded for group II (741.2 μm), and the lowest microleakage mean value was recorded for group V (133.2 μm). For the cementum-located restorations (sections A0 and B0), the highest mean value of MLKd was recorded for group I (851.6 μm), and the lowest value was recorded for group III (67.2 μm) (Figure 3, Table S1).

For the enamel-located restorations (sections A1 and B1), the highest mean value of MLKe was recorded for group V (581.8 μm), and the lowest microleakage mean value was recorded for group I (0 μm) (Figure 3, Table S1). For the cementum-located restorations (sections A0 and B0), the highest MLKe mean value was recorded for group IV (330 μm), and the lowest value was recorded for group II (21.6 μm) (Figure 3, Table S1).

### 3.2. Total Adhesive Interface Measurements

Total adhesive interface dimensions (AIL) showed non-significant differences between groups with only a few exceptions. A significantly increased AIL was observed for A1 sections of group V versus group III (*p* = 0.0285) and for A0 sections of group V versus groups I, II and III (*p* = 0.0317, *p* = 0.0266 and *p* = 0.0382, respectively) (Table S1).

The highest mean value of AIL was recorded for group V (3614 μm), and the lowest mean value was recorded for group III (2434 μm) (Figure 3, Table S1).

### 3.3. The Microleakage Ratio

The comparative statistical analysis of the values of the cervical microleakage ratio between groups showed no significant differences for both types of cavities except for for B1 sections where cervical microleakage ratio was significantly increased for group IV—GE versus group V-AX and the occlusal microleakage ratio for group V—AX versus group I—AX B (*p* = 0.0159 and *p* = 0.0476, respectively) (Table S1).

The intragroup comparisons of the cervical microleakage ratio between cementum-located and enamel-located restorations showed no differences. The intragroup comparisons of occlusal microleakage between cementum-located and occlusal-located restorations also showed non-significant differences except for the group V where an increased occlusal microleakage was calculated for A1 sections versus A0 sections (*p* = 0.0428) (Table S1).

Correlation analysis showed in group I—AX B, and strong positive correlations between MLKd and MLKe and between MLKe and AIL, were identified for some sections of cementum-located restorations (A0, B0), while for other sections (A0) a strong negative correlation between MLKe and AIL was observed. In group II—AF, there was a strong negative correlation between MLKd and AIL for cementum-located restorations (A0 sections) and a strong positive correlation between MLKe and AIL for enamel-located restorations (A1 sections). In group III—ES, for some samples of enamel-located restorations (B1), a strong negative correlation was identified between MLKd and MLKe (*p* < 0.05). For enamel-located restorations (A1, B1 sections) and cementum-located restorations (B0 sections) in group IV—GE, strong negative correlations were observed between MLKd and AIL. In group V—AX, strong positive correlations were identified for some samples of cementum-located and enamel-located restorations (A0, B0 and A1 sections) between MLKe and AIL and for other samples (B1 and B0) between MLKd and AIL. A strong negative correlation between MLKd and MLKe was observed for some samples of cementum-located restorations (A0), but for other samples (B0) a strong positive correlation was identified (Table S1).

## 4. Discussion

The present in vitro study used two ormocer materials and two conventional resin composites to restore class II cavities on extracted teeth and evaluated the adhesive performance of the materials by calculating the microleakage length ratio at restoration–tooth interface by dye penetration. Two restoration techniques where used.

The optimal adhesion of direct class II restorations to the dental structures depends on many factors [22,23,24,25,26,27] and is essential for providing good clinical outcomes and prevent restoration failure [7] or periodontal problems [12,28].

In the present study, dye penetration method was used as a microleakage test because it is the most frequently reported method, is easily reproducible [29] and is a good predictor of the clinical outcome of a restorative material [30]. Moreover, ISO standard No.11405/mentions this type of investigation, which has otherwise been used by recently published investigations [10,14,31].

All the restoration groups investigated in this study present microleakage, which is in accordance with other research studies [14,19,32]. The present study could not demonstrate the superiority of ormocer materials relative to conventional composites. Therefore, these results failed to reject the null hypothesis. There is an important variability of the data that may be associated with structural differences of dental structures and to marginal failures due to material dislocation during tooth sectioning. One of the limitations of the present study is the lack of control of dental substrate.

The relative equal performance of the materials may be due to the fact that the first generation of ormocer materials used in this study contain conventional methacrylate diluents, which may prevent to fulfil the expected potential [33,34], but similar shear bond strength [35], in vivo sealing ability and polymerization shrinkage are used [11]. The very recent dimethacrylate-diluent-free ormocer matrices seemed to improve ormocer behavior [7].

Except for a few sections, the restorative materials showed no significant differences of the adhesive performances in cervical areas irrespective the restoration type (cementum- and enamel-located) as revealed by the values of cervical microleakage ratio (Table S1). Other studies reported an increased microleakage associated with cervical margins located in cementum in comparison with the margins located in enamel [36,37]. This may be due to an inferior adhesion to cervical cementum of cervical-located restorations [38].

Additionally, almost no significant differences between tested materials in microleakage ratio were calculated for the occlusal areas of the restorations (Table S1). Overall, no significant differences between MLKd and MLKe ratio were calculated irrespective of dental material or restorative technique (Table S1), which is different from the results of other studies [1,38,39,40].

Our findings are in accordance with some studies reporting that bulk fill restorations have similar clinical performances as the restorations applied through the conventional layering technique [41,42,43], as revealed by intergroup comparisons between group I—AX B and other experimental groups (Table S1). Other studies provided contradictory results when comparing bulk-fill or layering techniques [44,45,46].

It is difficult to quantify the clinical impact of our findings, especially when all experimental materials are commercially available and already tested. Our study showed that microleakage around resin-based restorations is a certainty that hardly could be avoided, even under ideal laboratory conditions controlling many influencing variables. The present study suggests the clinical use of both material types that have remarkable properties. The negative correlations between MLKd and MLKe in some of the groups (I—AX B, III—ES and V—AX) highlighted by this study may be due to the vulnerability of cervical adhesion in comparison with the occlusal one, due to the minimum presence or the absence of enamel in the cervical area, which weakens the dentin adhesion [45]. This facilitates the fracture of the cervical adhesive interface during polymerization shrinkage [4,5]. One the other hand, the positive correlations between MLKd and AIL or MLKe and AIL in some sections (groups I—AX B, II—AF and V—AX) express the idea that the length of the microleakage is proportional to the length of the total adhesion layer. Part of the localized failures of the adhesive interface are certainly “true” microleakages produced mainly due to polymerization shrinkage stress [4]. A proportion of the microleakage could be the consequence of tooth sectioning that can brutally interrupt the adhesive interface [47]. Adhesion of biomaterials on hard and soft oral tissues is highly influenced by material composition [48].

## 5. Conclusions

Our study observed a relative uniform adhesive behavior of ormocer materials and conventional resin composites, which recommends both classes of dental materials to restore posterior teeth. All investigated materials had a comparable adhesive performance on both cervical and occlusal areas. Generally, the bulk fill ormocer material performed equally well when placed with bulk fill or proximal wall technique.

## Figures and Tables

**Table 1:** Compositions according to product brochures [15,16,17].

Type of Resin Composite	Restorative Material	Manufacturer	Matrix Monomers	Filler Content	Adhesive System
Ormocer Nano-hybrid	Admira Fusion X-tra^®^—AX	Voco, Cuxhaven Germany	-aliphatic and aromatic dimethacrylates, -methacrylate functionalized polysiloxane	84% weight; barium–aluminium–glass, pyrogenic silica dioxide	Futurabond M+^®^ (Voco) (1-step self-etching) -UDMA -HEMA -acid adhesive monomer -phosphorylate monomer -catalyzer -ethanol -water
Ormocer Nano-hybrid	Admira Fusion^®^—AF	Voco, Cuxhaven Germany	-aliphatic and aromatic dimethacrylates, -methacrylate functionalized polysiloxane	84% weight; barium–aluminium–glass, pyrogenic silica dioxide	
Microfilled-hybrid	Essentia^®^—ES	GC EUROPE N.V. Leuven	-UDMA, -Bis-MEPP, -Bis-EMA, -Bis-GMA, -TEGDMA	81% weight; pre-polymerized filler, barium glass, fumed silica	G-Bond^®^ (GC Corporation) (1-step self-etching) −4-META -UDMA -TEGDMA -distilled water -acetone
Microfilled-hybrid	G-aenial posterior^®^—GE	GC EUROPE N.V. Leuven	-UDMA -dimethacrylate co-monomers;	76% weight; pre-polymerized fillers 16–17 μm: silica-containing, strontium and lanthanoid fluoride containing inorganic filler >100 nm: silica, Inorganic filler <100 nm: fumed silica;	

UDMA = urethane dimetacrylate (Sigma-Aldrich Chemie GmbH, Steinheim, Germany); Bis-GMA = -bisphenol glycidyl dimethacrylate (Sigma-Aldrich); TEGDMA = triethyleneglycol—dimethacrylate (Sigma-Aldrich); Bis-MEPP = 2,2-Bis(4-methacryloxypolyethoxyphenyl) propane (Sigma-Aldrich); Bis-EMA = 2,2-bis[4-(2-hydroxy-3-methacryloxypropoxy)phenyl] propane (Sigma-Aldrich); HEMA = hydroxyethylmethacrylate (Sigma-Aldrich); 4-META = 4-methacryloyloxyethyl trimellitate anhydride (Sigma-Aldrich).

## Data Availability

The datasets used and/or analyzed during the current study are available from the corresponding authors on reasonable request.

## Supplementary Materials

The following are available online at https://www.mdpi.com/article/10.3390/ma14154299/s1, Figure S1: Steps in sample preparation. Cavities on teeth of groups I—V (A); Varnish application on teeth of group I—AXB (B); Immersion in dye solution of group I—AXB (C); Microtome sectioning of a tooth from group I—AXB (D); Sections of group I—AXB (E). Table S1: Mean values and standard deviation (SD) of evaluated parameters as well as the significance (*p* values) of the recorded differences.
