# Supplementary material for: In Vitro Study on the Adhesive Performance of Some Resin-Based Materials Used to Restore Class II Cavities
Source: Materials (Basel). 2021 Jul 31;14(15):4299. doi: 10.3390/ma14154299 (PMC8348953; doi:10.3390/ma14154299)
Supplement: Supplementary file 1 [file materials-14-04299-s001.zip › materials-1325751-supplementary.pdf]

materials

MDPI

Table S1. Mean values and standard deviation (SD) of evaluated parameters as well as the significance (*p* values) of the recorded differences.

A.Microleakage in cervical area

Group	Section	Mean	±	SD	*p-value*								
I	A1	679.4	±	885.92	A1	I-II	> 0.9999	B1	I-II	0.4683	I	A1-A0	0.625
	A0	357	±	360.06		I-III	> 0.9999		I-III	0.5238		B1-B0	0.3125
	B1	591.8	±	688.66		I-IV	0.7857		I-IV	0.7266		A1-B1	0.75
	B0	851.6	±	894.48		I-V	0.6349		I-V	0.3651		A0-B0	0.4375
II	A1	741.2	±	717.23		II-III	0.662		II-III	0.3333	II	A1-A0	0.625
	A0	424.4	±	636.16		II-IV	0.7698		II-IV	0.0994		B1-B0	0.875
	B1	342	±	253.04		II-V	0.2259		II-V	0.2381		A1-B1	0.2475
	B0	430	±	596.15		III-IV	0.8235		III-IV	0.0476*		A0-B0	> 0.9999
III	A1	556.2	±	562.43		III-V	0.3382		III-V	0.8413	III	A1-A0	0.125
	A0	67.2	±	92.41		IV-V	0.1394		IV-V	0.0317*		B1-B0	0.625
	B1	210	±	394.52	A0	I-II	> 0.9999	B0	I-II	0.3333		A1-B1	0.125
	B0	429.8	±	353.62		I-III	0.2857		I-III	0.746		A0-B0	0.125
IV	A1	628.6	±	420.73		I-IV	0.958		I-IV	0.4127	IV	A1-A0	0.1245
	A0	345.4	±	313.4		I-V	0.6419		I-V	0.3016		B1-B0	0.0625
	B1	718.8	±	374.72		II-III	0.6825		II-III	0.8571		A1-B1	0.4969
	B0	322.6	±	398.63		II-IV	0.6825		II-IV	> 0.9999		A0-B0	0.8125
V	A1	281.8	±	69.31		II-V	0.5397		II-V	> 0.9999	V	A1-A0	0.293
	A0	470.8	±	384.21		III-IV	0.1764		III-IV	0.6905		B1-B0	0.25
	B1	133.2	±	197.74		III-V	0.0476*		III-V	0.2765		A1-B1	0.3125
	B0	208.2	±	224.18		IV-V	0.5872		IV-V	0.8254		A0-B0	0.3364

B.Microleakage in occlusal area

Group	Section	Mean	±	SD	*p-value*								
I	A1	342.6	±	422.58	A1	I-II	0.7315	B1	I-II	0.4444	I	A1-A0	0.125
	A0	46.4	±	103.75		I-III	0.1667		I-III	0.4444		B1-B0	> 0.9999
	B1	0.00	±	0.00		I-IV	> 0.9999		I-IV	0.1474		A1-B1	0.1441
	B0	81	±	181.12		I-V	0.4475		I-V	0.0476*		A0-B0	> 0.9999
II	A1	264.4	±	252.1		II-III	0.5238		II-III	0.8413	II	A1-A0	0.8815
	A0	281.6	±	226.73		II-IV	0.8254		II-IV	0.5238		B1-B0	0.5
	B1	100.8	±	162.02		II-V	0.254		II-V	0.1746		A1-B1	0.25
	B0	21.6	±	48.3		III-IV	0.5238		III-IV	0.5238		A0-B0	0.125
III	A1	123.2	±	275.48		III-V	0.127		III-V	0.3333	III	A1-A0	> 0.9999
	A0	40.2	±	89.89		IV-V	0.3016		IV-V	0.7186		B1-B0	> 0.9999
	B1	167.8	±	278.87	A0	I-II	0.0873	B0	I-II	> 0.9999		A1-B1	0.5
	B0	207.4	±	289.1		I-III	> 0.9999		I-III	0.4444		A0-B0	0.5
IV	A1	260.6	±	243.46		I-IV	> 0.9999		I-IV	0.2857	IV	A1-A0	0.5
	A0	125.6	±	280.85		I-V	0.2857		I-V	0.7222		B1-B0	0.7874
	B1	291.2	±	363.11		II-III	0.0873		II-III	0.4444		A1-B1	> 0.9999
	B0	330	±	367.13		II-IV	0.2857		II-IV	0.1667		A0-B0	0.25
V	A1	581.8	±	519.55		II-V	0.6905		II-V	0.4444	V	A1-A0	0.125
	A0	262	±	369.02		III-IV	> 0.9999		III-IV	0.5238		B1-B0	0.375
	B1	379.6	±	385.3		III-V	0.2857		III-V	0.6825		A1-B1	0.428
	B0	145.6	±	203.69		IV-V	0.4048		IV-V	0.5238		A0-B0	0.5

C.Total adhesive interface

Group	Section	Mean	±	SD	p-value								
I	A1	3017.4	±	313.65	A1	I-II	0.4375	B1	I-II	0.0362	I	A1-A0	0.625
	A0	2769.8	±	474.53		I-III	0.3347		I-III	0.0958		B1-B0	0.8529
	B1	3079.6	±	342.64		I-IV	0.6048		I-IV	0.5714		A1-B1	0.8529
	B0	3132.4	±	493.46		I-V	0.0667		I-V	0.0652		A0-B0	0.3125
II	A1	2798.6	±	320.9		II-III	0.8413		II-III	0.5405	II	A1-A0	> 0.9999
	A0	2825.4	±	348.82		II-IV	> 0.9999		II-IV	0.4595		B1-B0	0.8469
	B1	2643	±	182.97		II-V	0.125		II-V	0.0012**		A1-B1	0.1875
	B0	2584	±	603.96		III-IV	0.8547		III-IV	0.3146		A0-B0	0.5361
III	A1	2760.2	±	464.22		III-V	0.0285*		III-V	0.0119*	III	A1-A0	0.4756
	A0	2933.8	±	270.48		IV-V	0.0852		IV-V	0.0767		B1-B0	0.3227
	B1	2433.6	±	684.4	A0	I-II	0.8413	B0	I-II	0.1545		A1-B1	0.0319*
	B0	2665.6	±	372.43		I-III	0.8413		I-III	0.1298		A0-B0	0.1295
IV	A1	2831.6	±	704.71		I-IV	> 0.9999		I-IV	0.0556	IV	A1-A0	0.3903
	A0	3010.4	±	696.32		I-V	0.0317*		I-V	0.9022		B1-B0	0.1875
	B1	2886	±	648.62		II-III	0.5979		II-III	0.8036		A1-B1	0.8439
	B0	2604.2	±	402.66		II-IV	0.6097		II-IV	> 0.9999		A0-B0	0.0625
V	A1	3614	±	545.14		II-V	0.0266*		II-V	0.1556	V	A1-A0	0.0956
	A0	3510.6	±	444.43		III-IV	0.8244		III-IV	0.8125		B1-B0	0.0958
	B1	3570	±	382.43		III-V	0.0382*		III-V	0.1208		A1-B1	0.7951
	B0	3096	±	410.66		IV-V	0.2127		IV-V	0.2222		A0-B0	0.1577

D.Microleakage ratio in cervical area

Group	Section	Mean	±	SD	p-value								
I	A1	0.23	±	0.3	A1	I-II	0.8333	B1	I-II	0.5844	I	A1-A0	> 0.9999
	A0	0.15	±	0.18		I-III	0.8333		I-III	0.5238		B1-B0	0.5617
	B1	0.2	±	0.25		I-IV	0.6349		I-IV	0.5864		A1-B1	0.75
	B0	0.24	±	0.24		I-V	0.6349		I-V	0.2857		A0-B0	0.5952
II	A1	0.28	±	0.27		II-III	0.6753		II-III	0.3333	II	A1-A0	0.625
	A0	0.14	±	0.21		II-IV	0.9452		II-IV	0.1644		B1-B0	0.875
	B1	0.13	±	0.1		II-V	0.1774		II-V	0.4032		A1-B1	0.2537
	B0	0.16	±	0.23		III-IV	0.7081		III-IV	0.0952		A0-B0	> 0.9999
III	A1	0.21	±	0.21		III-V	0.2263		III-V	0.8413	III	A1-A0	0.125
	A0	0.02	±	0.03		IV-V	0.1551		IV-V	0.0159*		B1-B0	0.625
	B1	0.09	±	0.16	A0	I-II	> 0.9999	B0	I-II	0.3333		A1-B1	0.125
	B0	0.17	±	0.14		I-III	0.2857		I-III	0.5699		A0-B0	0.125
IV	A1	0.27	±	0.24		I-IV	0.8186		I-IV	0.4127	IV	A1-A0	0.1571
	A0	0.13	±	0.12		I-V	0.8927		I-V	0.1645		B1-B0	0.125
	B1	0.28	±	0.2		II-III	0.6825		II-III	0.6825		A1-B1	0.8976
	B0	0.13	±	0.17		II-IV	0.5556		II-IV	> 0.9999		A0-B0	> 0.9999
V	A1	0.08	±	0.02		II-V	0.5397		II-V	> 0.9999	V	A1-A0	0.2602
	A0	0.14	±	0.11		III-IV	0.1746		III-IV	0.6905		B1-B0	0.25
	B1	0.03	±	0.05		III-V	0.0952		III-V	0.1647		A1-B1	0.1875
	B0	0.06	±	0.07		IV-V	0.898		IV-V	0.8254		A0-B0	0.3654

E. Microleakage ration at occlusal area

Group	Section	Mean	±	SD	p-value								
I	A1	0.11	±	0.13	A1	I-II	0.1667	B1	I-II	0.4444	I	A1-A0	0.125
	A0	0.02	±	0.05		I-III	0.9231		I-III	0.4444		B1-B0	> 0.9999
	B1	0.00	±	0.00		I-IV	0.9232		I-IV	0.1432		A1-B1	0.1271
	B0	0.02	±	0.05		I-V	0.6641		I-V	0.0476*		A0-B0	> 0.9999
II	A1	0.09	±	0.09		II-III	0.5238		II-III	0.8413	II	A1-A0	0.7137

	A0	0.11	±	0.1		II-IV	0.8425		II-IV	0.5238		B1-B0	0.5
	B1	0.04	±	0.06		II-V	0.4093		II-V	0.3333		A1-B1	0.25
	B0	0.01	±	0.02		III-IV	0.4048		III-IV	0.5238		A0-B0	0.125
III	A1	0.05	±	0.1		III-V	0.1667		III-V	0.3333	III	A1-A0	> 0.9999
	A0	0.01	±	0.03		IV-V	0.5685		IV-V	0.9309		B1-B0	> 0.9999
	B1	0.07	±	0.11	A0	I-II	0.127	B0	I-II	> 0.9999		A1-B1	0.5
	B0	0.08	±	0.11		I-III	> 0.9999		I-III	0.4444		A0-B0	0.5
IV	A1	0.11	±	0.11		I-IV	> 0.9999		I-IV	0.5	IV	A1-A0	0.25
	A0	0.05	±	0.12		I-V	0.4048		I-V	0.7222		B1-B0	0.644
	B1	0.11	±	0.14		II-III	0.0873		II-III	0.4444		A1-B1	0.8746
	B0	0.14	±	0.16		II-IV	0.2857		II-IV	0.1667		A0-B0	0.25
V	A1	0.15	±	0.12		II-V	0.5226		II-V	0.4444	V	A1-A0	0.0428*
	A0	0.07	±	0.09		III-IV	> 0.9999		III-IV	0.5238		B1-B0	0.375
	B1	0.11	±	0.1		III-V	0.2857		III-V	0.6825		A1-B1	0.4638
	B0	0.04	±	0.06		IV-V	0.5238		IV-V	0.5238		A0-B0	0.75

*p<0.05; **p<0.01; ***p<0.001. A1, B1= part of the sections containing enamel-located restorations; A0, BO= part of the sections containing cementum-located restorations.
